# Nanohybrid-based immunosensor prepared for *Helicobacter pylori* BabA antigen detection through immobilized antibody assembly with @ Pd_nano_/rGO/PEDOT sensing platform

**DOI:** 10.1038/s41598-020-78068-w

**Published:** 2020-12-04

**Authors:** Shaivya Gupta, Utkarsh Jain, Bayu Tri Murti, Athika Darumas Putri, Ashutosh Tiwari, Nidhi Chauhan

**Affiliations:** 1 Amity Institute of Nanotechnology, Amity University, Noida, Uttar Pradesh 201303 India; 2 Department of Chemistry, Durban University of Technology, Durban, 4000 South Africa; 3 Semarang College of Pharmaceutical Sciences, Jl. Letnand Jendral Sarwo Edi Wibowo, Semarang City, 50192 Indonesia; 4 Institute of Advanced Materials, IAAM, Gammalkilsvägen 18, 590 53 Ulrika, Sweden; 5 VBRI, 7/16 Kalkaji Extn., New Delhi, 110 019 India

**Keywords:** Biological techniques, Cancer, Computational biology and bioinformatics, Gastroenterology, Health care, Materials science, Nanoscience and technology

## Abstract

The gastric colonization of human hosts by *Helicobacter pylori (H. pylori)* increases the risk of developing gastritis, ulcers and gastric cancer. To detect *H. pylori,* a nanohybrid-based BabA immunosensor is developed herein. BabA is an outer membrane protein and one of the major virulence factors of *H. pylori*. To design the immunosensor, an Au electrode is loaded with palladium nanoparticles (Pd_nano_) by electrodeposition to generate reduced graphene oxide (rGO)/poly(3,4-ethylenedioxythiophene) (PEDOT). The immobilization of these nanostructured materials imparts a large surface area and electroconductivity to bio-immune-sensing molecules (here, the BabA antigen and antibodies). After optimization, the fabricated immunosensor has the ability to detect antigens *(H. pylori)* in a linear range from 0.2 to 20 ng/mL with a low LOD (0.2 ng/mL). The developed immunosensor is highly specific, sensitive and reproducible. Additionally, in silico methods were employed to better understand the hybrid nanomaterials of the fabricated Pd_nano_/rGO/PEDOT/Au electrode. Simulations performed by molecular docking, and Metropolis Monte Carlo adsorption studies were conducted. The results revealed that the hybrid nanomaterials exhibit a stable antigen–antibody complex of BabA, yielding the lowest binding energy in relation to the electrode materials, emphasizing the functionality of the constructed electrodes in the electrochemical immunosensor.

## Introduction

*Helicobacter pylori* (*H. pylori*) prevalence in hosts contributes to chronic gastritis and gastric cancer. Gram-negative bacterium is the type of *H. pylori* that ultimately leads to the progression of ulcers and cancer by infecting the gastric mucosa^1–3^. *H. pylori* develops mechanisms for gastric colonization with the help of virulence factors, causes gastric ulceration and plays a major role in the progression of stomach cancer. Collectively, with *H. pylori* colonization, the inflammatory response of the host and the interaction of environmental and dietary factors are a few of the major causes of gastric diseases^4^. The most likely transmission route is individual-to-individual, but fecal–oral transmissions were also identified^5–7^. It was observed that the death rate due to stomach cancer caused by *H. pylori* colonization has been increasing significantly, and therefore, an immunosensor was stepwise fabricated for detecting *H. pylori* specific antigens in biological samples.

In the first step, studies were performed through molecular docking. Molecular docking was incorporated to predict the modes of the interactions of the nanocomposites with each other and the nanocomposite with the *H. pylori* biomarker. In this work, emerging computational methods such as molecular docking, Metropolis Monte Carlo (MMC), and binding energy calculations were employed to validate the energetic parameters of the immunosensor. The results revealed the adsorption behavior of the BabA antigen and its antibody complexes towards a layer-by-layer surface. Moreover, 3D structures of antigen (Ag) and antibody (Ab) biomolecules were used for docking simulations to investigate the BabA Ag–Ab interactions. The best docked model is subsequently elaborated with the hybrid electrode materials (i.e., Pd_nano_/rGO/PEDOT/Au) to mimic the experimental process. *H. pylori* BabA is used as a biomarker^8^. *H. pylori* antigen binding adhesin (BabA) protein is one of the major virulence factors of molecular docking^9–11^. Strains expressing BabA have shown a high risk of peptic ulceration and development of gastric diseases. In addition, BabA binding to host cells triggers translocation of CagA protein and promotes the pathogenicity of VacA protein^12^. Recently, because of their intrinsic properties, such as the antibody–antigen interactions determined by directly observation, electrochemical immunosensors have attracted considerable attention^13^. Thus, the requirements of the nanocomposite used in an immunosensor are very stringent: the nanocomposite should be a very good carrier and all together should not obstruct electron exchange or append proteins; the nanomaterial should have active sites and a vast surface area to immobilize many antibodies^14^.

Standard methods available for *H. pylori* detection are either invasive or noninvasive. Invasive procedures comprise histology, rapid urease tests^15^, polymerase chain reaction^16^, microbiological culture^17^ and biopsy-based tests. Noninvasive practices incorporate stool antigen tests^18^, serology and urea breath tests^19^. These methods, however, are time consuming, expensive and have a short shelf-life. Thus, to surmount all these downsides, immunosensors have been considered to be better because of their low LOD, high selectivity, fast response and ease of handling. Despite the numerous advancements that have been made, there is an unprecedented need for a sensor that is based on highly selective antigen–antibody interactions and exhibits good sensitivity.

**Scheme 1:**
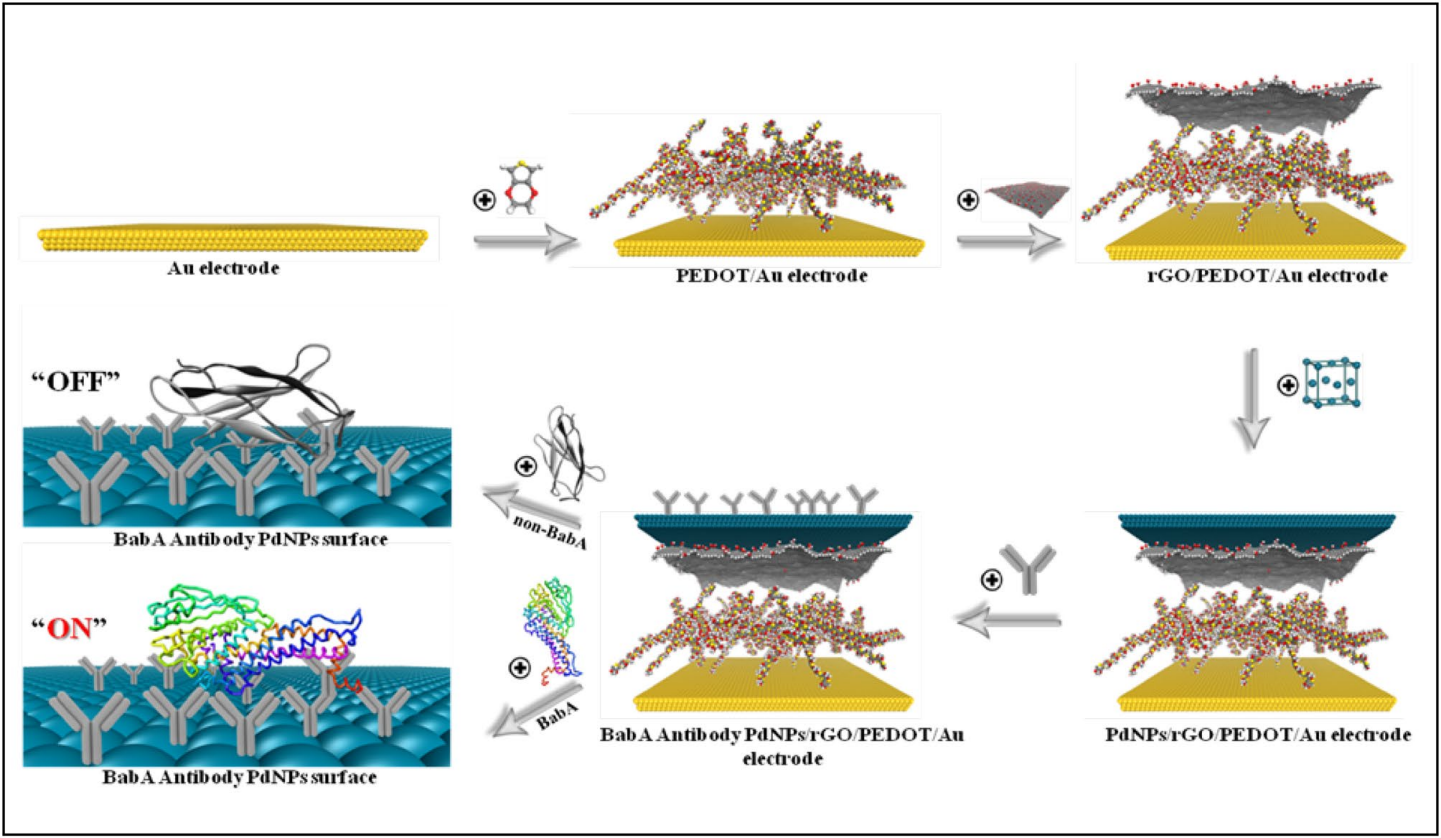
Schematic illustration of the steps involved in the preparation of the BabA Ab@Pd_nano_/rGO/PEDOT/Au electrode. The figures were obtained from Material Studio v.16.1.0.21 (BIOVIA, D. S. 2016. Material Studio modelling. v.16.1.0.21 ed. San Diego: Dassault Systemes) and Discovery Studio v.16.10.15350 software packages (BIOVIA, D. S. 2016. Discovery Studio modelling. v.16.10.15350 ed. San Diego: Dassault Systemes).

Herein, we have developed an electrochemical immunosensor using a hybrid nanomaterial probe with metal nanoparticles and a conducting polymer. The novel hybrid nanomaterials are synthesized and engineered for *H. pylori* detection with the incorporation of nanoscale properties, i.e., the large surface area and strong electrical conductivity of palladium nanoparticles (Pd_nano_), poly(3,4-ethylenedioxythiophene) (PEDOT) and reduced graphene oxide (rGO) with the BabA antigen. In addition, stability and biocompatibility are considered. Furthermore, noninvasive testing was conducted on spiked stool samples using this application*.* The goal of this study was to provide a promising platform for the early detection of the *H. pylori* antigen using label-free hybrid nanomaterial-based immunosensors (Scheme 1).

.

## Results and discussions

### Computational simulation and modeling

Computational simulations have been effectively used to observe the mechanism of nanocomposite construction and the interaction phenomena occurring in biosensor platforms^20,21^. In this work, the layer-by-layer electrode position method used to prepare the hybrid nanomaterials was successfully mimicked through computational modeling to reproduce the energetic contributions towards binding with the biomolecule complex. Because of the utilization of a large-scale substrate-adsorbate system, i.e., the adsorbate structures of the Ag-Ab complex as well as the substrates containing organic and inorganic nanostructures, the overall energy of each trajectory was computed with MMC. The binding energies were calculated according to Eq. (1), showing its tremendous computational capability.

### Binding energy calculation

The binding energy (*E*_*b*_) between the substrate and the adsorbate layers was computed according to the following equation:

1 $$E_{b} = \, E_{comp} - \, E_{ads} - \, E_{subs}$$

where *E*_*comp*_ = total energy of the complex; *E*_*ads*_ = interaction energy between the adsorbate; and *E*_*subs*_ = substrate energy.

**Figure 1:**
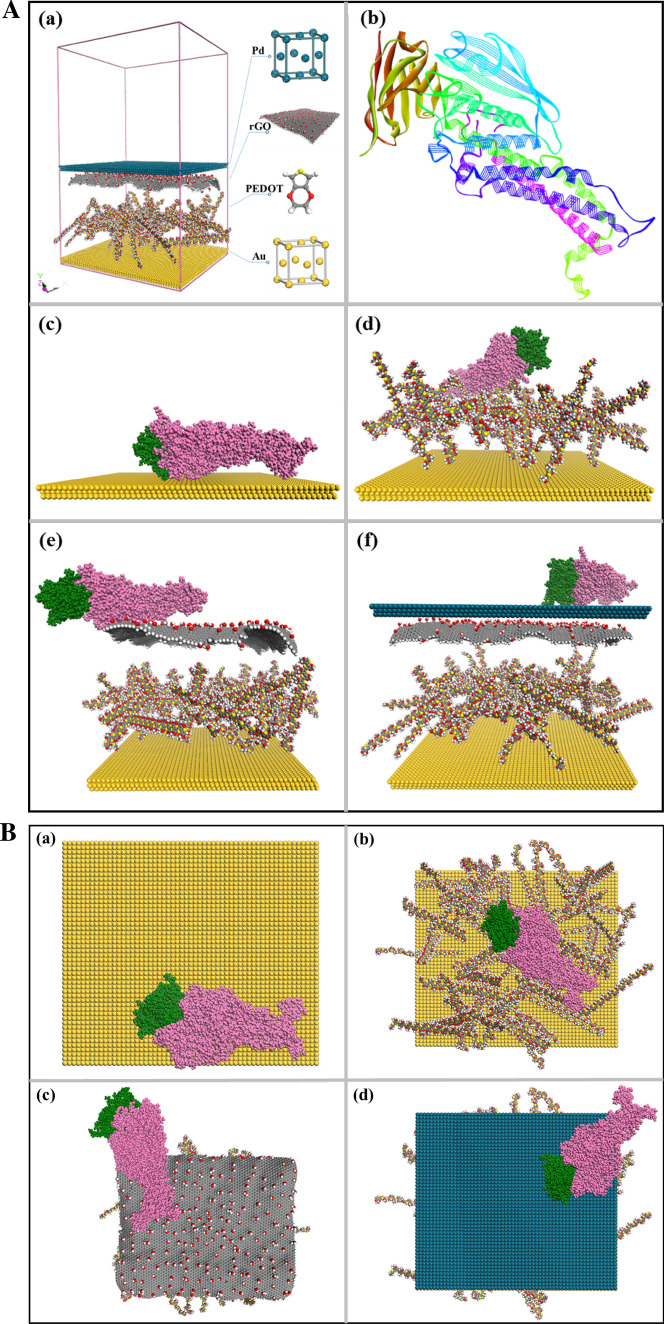
(**A**) Periodic boundary structure containing Pd_nano_/rGO/PEDOT/Au electrode along with the origin of each component (a) and docked structure of BabA Ag and its Ab. The Ag and Ab structures shown as line- and solid-ribbon depictions, respectively (b). The lowest energy configuration of Ag (BabA protein; green)-Ab (anti-BabA; purple) molecule onto Au electrode (c), PEDOT/Au electrode (d), rGO/PEDOT/Au electrode (e) and Pd_nano_/rGO/PEDOT/Au electrode (f). For the figures (c–f), the complex structures are depicted from front views using ball and stick models. The figures were obtained from Material Studio v.16.1.0.21 (BIOVIA, D. S. 2016. Material Studio modelling. v.16.1.0.21 ed. San Diego: Dassault Systemes) and Discovery Studio v.16.10.15350 software packages (BIOVIA, D. S. 2016. Discovery Studio modelling. v.16.10.15350 ed. San Diego: Dassault Systemes). (**B**) The lowest energy configurations of Ag (BabA protein; green)-Ab (anti-BabA; purple) molecule onto Au electrode (a), PEDOT/Au electrode (b), rGO/PEDOT/Au electrode (c) and Pd_nano_/rGO/PEDOT/Au electrode (d). The depictions are undertaken at the top views format. The figures were obtained from Material Studio v.16.1.0.21 (BIOVIA, D. S. 2016. Material Studio modelling. v.16.1.0.21 ed. San Diego: Dassault Systemes) and Discovery Studio v.16.10.15350 software packages (BIOVIA, D. S. 2016. Discovery Studio modelling. v.16.10.15350 ed. San Diego: Dassault Systemes).

The overall energies of the reactant (i.e., layer-by-layer substrates) and the final products (i.e., substrate-adsorbate complexes) were determined with the binding energy calculations by classic and pragmatic approaches^22^. The whole energies were defined according to Eq. (2).

2 $$E_{tot} = \, E_{val} - \, E_{nb}$$

where the valence energy (*E*_*val*_) includes the bond, angle, torsion and inversion energies, while the nonbond energy (*E*_*nb*_) includes the van der Waals, long-range correction, and electrostatic energies. A reasonable periodic cell size was employed to demonstrate the appropriate surface area. Since the hybrid nanomaterial probe contains four material elements (Au, PEDOT, rGO and Pd_nano_), we decided to carry out the simulations with respect to each substrate surface (Au, PEDOT/Au, rGO/PEDOT/Au and Pd_nano_/rGO/PEDOT/Au) (Fig. 1A (a)) and thoroughly examined their binding energy differences with respect to the Ag–Ab complex.

Prior to the MMC studies, the interaction of Ag and Ab was defined through docking simulation by employing ZDOCK and ZRANK as scoring functions. The best pose used for further calculation was Pose-24, since it indicated the presence of the important amino acid residues of the Ab binding surface (i.e., Gly191, Ser234, and Ser244)^23^ among the top thirty binding poses. The results and scoring data are summarized in Table 1. As a preliminary step, the Ag and Ab structures were derived from PDB codes and treated using CHARMM19 for geometry optimization. The optimized structures of Ab and Ag were introduced as the receptor and ligand for docking analysis, respectively, resulting in approximately 2000 docked poses, and the best docked model, which fulfilled the active binding region of Ab, was selected from the largest clusters to perform further interaction studies with the nanomaterial surfaces (Fig. 1A (b)).

**Table 1:** The docking score of the selected pose.

Pose	Rank	Density	Cluster Size	ZDOCK Score	ZRANK Score
Pose1	1	14	21	18.38	− 88.566
Pose2	2	46	56	15.50	− 86.315
Pose3	3	3	1	15.72	− 83.160
Pose4	4	19	20	15.24	− 82.161
Pose5	5	24	30	15.18	− 81.974
Pose6	6	43	45	15.30	− 81.576
Pose7	7	45	56	16.38	− 79.065
Pose8	8	14	15	16.34	− 77.213
Pose9	9	3	1	14.96	− 76.174
Pose10	10	37	56	15.38	− 75.623
Pose11	11	6	11	15.08	− 74.304
Pose12	12	48	56	15.30	− 73.720
Pose13	13	9	13	16.34	− 73.448
Pose14	14	21	31	14.78	− 72.917
Pose15	15	5	5	16.26	− 72.672
Pose16	16	2	5	16.86	− 71.257
Pose17	17	5	5	16.04	− 70.912
Pose18	18	42	44	14.84	− 70.469
Pose19	19	28	44	15.82	− 70.273
Pose20	20	3	1	15.76	− 70.005
Pose21	21	66	70	15.22	− 69.889
Pose22	22	16	16	17.84	− 69.636
Pose23	23	24	30	15.04	− 69.604
**Pose24**	**24**	**6**	**13**	**15.32**	**− 69.412**
Pose25	25	20	21	14.84	− 69.296
Pose26	26	28	29	16.42	− 68.789
Pose27	27	3	1	15.16	− 68.446
Pose28	28	27	30	15.90	− 68.257
Pose29	29	27	29	15.82	− 68.158
Pose30	30	18	20	15.00	− 68.140

MMC simulation is a reliable approach for predicting adsorption. During the docking studies, MMC simulation was carried out on each substrate layer, which was followed by binding energy observation. By performing the calculation of single point energy, the energetics of each composite was observed and correlated with those of other materials. The optimized configuration of the whole system is depicted in Fig. 1A (c–f),B (a–d), which comprise different views, while the generated values of the binding energy are depicted in Table 2. The negative given values/binding scores demonstrate stable complex formation during the interaction studies. Indeed, the Au surface produces a direct efficient binding energy after the adsorption of the protein complex onto the electrode surface, suggesting that the metallic properties induce a physical interaction between the Au surface and the biomolecule^24^. On the other hand, the addition of the PEDOT polymers tends to weaken the binding interaction of the complex, which was shown by the significant increase in the binding energy to − 374.74 kJ/mol (Fig. 1A(d),B (c)). Similar evidence was shown after rGO assembled on the biosurface. The lowest binding energy was found for the fully packed system composed of Pd_nano_/rGO/PEDOT/Au with a binding energy of − 1386.59 kJ/mol (Fig. 1A(f),B (d)). However, the other surfaces showed higher binding energies (weaker binding interactions). Thus, the complete layered substrate efficiently releases more energy than the others. In addition, the presence of PEDOT, rGO, and Pd_nano_ on the Au electrode contributes to the low binding energy of the complete system (i.e., strengthening the interaction with the analyte). This further suggests that assembling the hybrid nanomaterials in the immunosensing system results in the preferable interaction between the corresponding complex and analyte. Indeed, the results calculated with this experimental strategy and previous theoretical studies have an agreeable outcome, indicating that combining Pd_nano_ with rGO can possibly improve the sensing performance to achieve a performance better than that obtained with Pd_nano_ with rGO individually due to their excellent conductivity. The synergy of the experimental and computational results is described herein.

**Table 2:** Binding energy values (in kJ/mol) of the *layer-by-layer* system against Ag–Ab complex.

Substrates	Total energy (kJ/mol)			
	*E*_*comp*_	*E*_*ads*_	*E*_*subs*_	*E*_*b*_
Au	− 1,272,451.94	− 13,422.60	− 1,257,723.08	− 1,306.26
PEDOT/Au	− 480,494.05	− 13,210.37	− 466,908.94	− 374.74
rGO/PEDOT/Au	2,310,114.18	− 13,140.72	2,323,558.48	− 303.58
Pd_nano_/rGO/PEDOT/Au	966,642.92	− 13,373.48	981,402.99	− 1386.59

### Nano-electrochemical deposition of Pd_nano_ over the rGO/PEDOT/Au electrode

**Figure 2:**
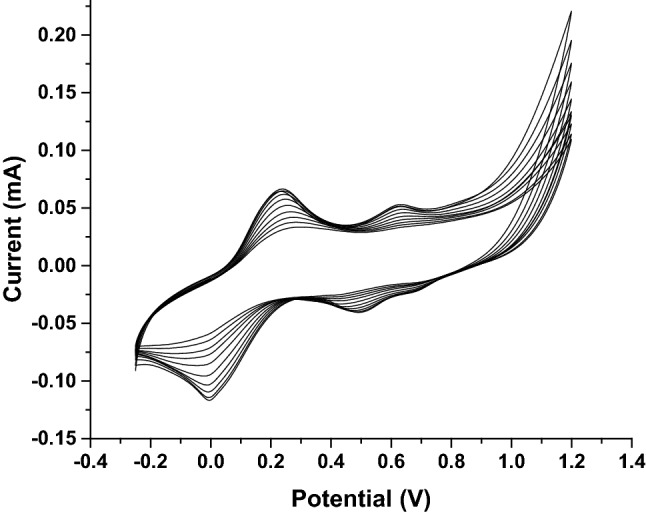
Pd_nano_ electrodeposition obtained with 5 mM [Fe(CN)_6_]^3−/4−^ electrolyte at 20 mV/s between potential range − 0.25 V to + 1.2 V for 10 cycles.

Figure 2 depicts the electrodeposition of Pd_nano_ over the rGO/PEDOT/Au electrode at the specified scan rate (50 mV/s) by adjusting the voltage from − 0.25 to + 1.2 V for 10 cycles. Ten cycles of CV were repeated for the electrodeposition of Pd_nano_ to achieve reproducible curves. In first few cycles, peaks were not differentiable because the added precursor was not completely dissolved. Here, CV demonstrates the characteristic current features of the decrease in Pd size at a voltage of 0.5 V, Pd oxide formation at 0.24 V and 0.62 V (at the same time), and hydrogen adsorption and desorption between 0.1 and − 0.1 V. Pd_nano_ accumulated on the modified electrode while the potential was scanned in the negative direction, and the peak at − 0.02 V indicates the process of reducing protons that are adsorbed on the Pd surface to hydrogen. The Pd_nano_ particles deposited on the surface of the electrode are further oxidized to Pd^2+^ to create a layer of Pd oxide (0.24 V)^25,26^. During the reverse potential scan, the size of the synthesized Pd oxides further decreased, leading to Pd_nano_ particles capable of hydrogen adsorption. The enhancement of peaks was observed during this repeated cycling process, confirming that the process of Pd_nano_ deposition on the Au electrode occurred. These characterizations clearly indicate the successive synthesis of a Pd_nano_ layer over the nanocomposite-modified Au electrode.

The established procedure used to reduce the PdCl_4_^2−^ complex to Pd is as follows:

$${\text{PdCl}}_{{4}}^{{{2} - }} + {\text{ 2e}}^{ - } \to {\text{ Pd }} + {\text{ 4Cl}}^{ - }$$

### Nanocharacterization of the various steps involved in the immunoassembly

#### Electron microscopic characterization of nanocomposites

**Figure 3:**
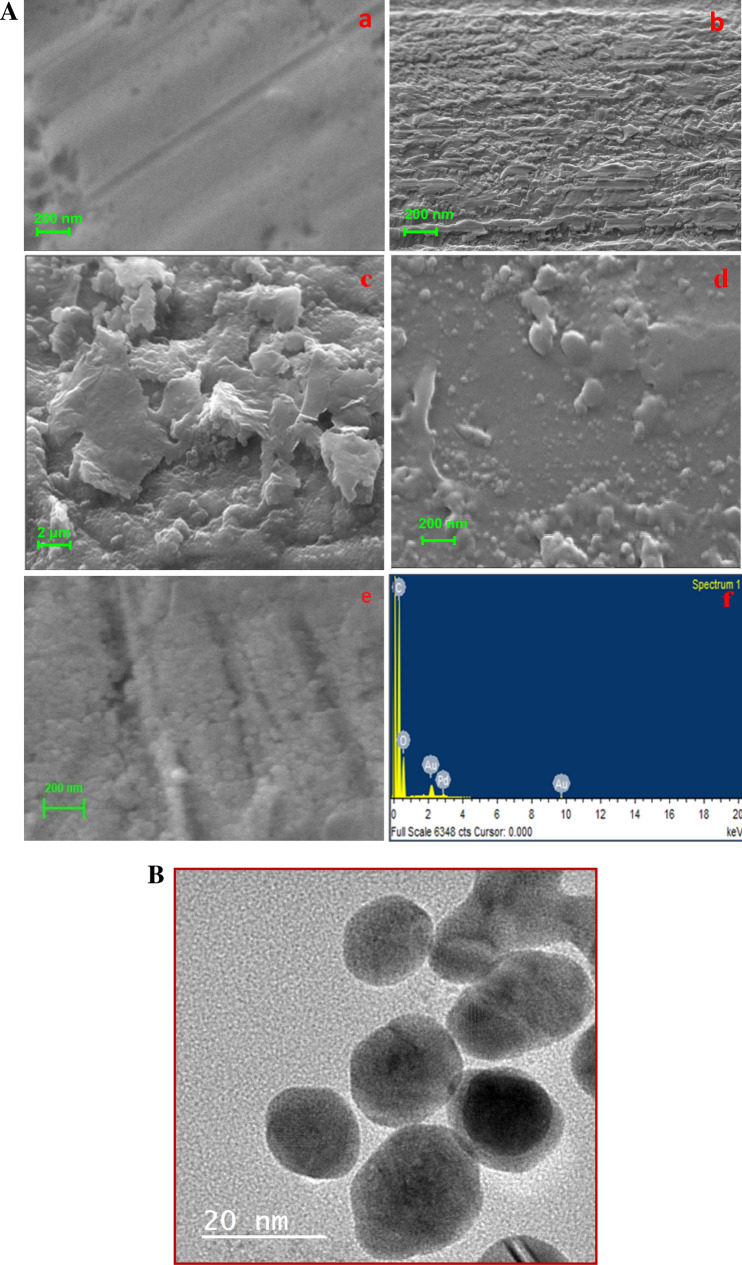
(**A**) SEM micrograph image of (a) bare Au electrode, (b) PEDOT/Au electrode, (c) rGO/PEDOT/Au electrode, (d) Pd_nano_/rGO/PEDOT/Au electrode, (e) BabA Ab@Pd_nano_/rGO/PEDOT/Au electrode and (f) EDX spectrum of Pd_nano_/rGO/PEDOT/Au electrode. (**B**) TEM image of Pd_nano_.

Figure 3A shows the morphological study results obtained by SEM for the (a) bare Au electrode and (b) PEDOT, (c) rGO/PEDOT, (d) Pd_nano_/rGO/PEDOT and (e) BabA Ab@Pd_nano_/rGO/PEDOT-modified Au electrode. The Au electrode surface was a smooth and uniform layer of a bare Au electrode (Fig. 3A (a)). Figure 3A (b) reveals the granular structure of PEDOT on the surface of the Au electrode, while the rGO film shows a wrinkled and crumpled surface (image c). As shown in Fig. 3A (d), a spherical structure with uniformly distributed nanospheres was observed for Pd_nano_ on the rGO/PEDOT-modified Au electrode surface. Pd_nano_ almost covered and was well distributed on the entire graphene surface of the rGO/PEDOT/Au electrode. Figure 3A (e) depicts the globule-like structure of BabA Ab, which formed clusters and was distributed evenly on the surface of the nanocomposite modified electrode. Figure 3A (f) shows the EDX spectrum of carbon, oxygen, gold and palladium. The composition of these elements in the spectrum (Au = 4.88%, C = 60.38%, O = 33.47%, and Pd = 1.27%) confirms that these elements are present in the nanocomposite-modified electrode.

#### Visualization of Pd_nano_ through transmission electron microscopy

Figure 3B shows transmission electron microscopy (TEM) images of nanoparticle clusters of palladium, showing their shapes and size distributions. Round or oval shaped particles with 20 nm sizes were determined.

#### The electrochemical characterizations of the amplified strategies

**Figure 4:**
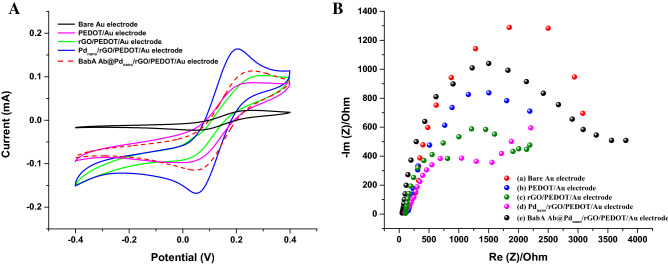
(**A**) CV curves of bare Au electrode, PEDOT/Au electrode, rGO/PEDOT/Au electrode, Pd_nano_/rGO/PEDOT/Au electrode and BabA Ab@ Pd_nano_/rGO/PEDOT/Au electrode obtained with 5 mM [Fe(CN)_6_]^3−/4−^ electrolyte containing 0.5 mM sodium phosphate buffer (pH 7.5) at 20 mVs^−1^. (**B**) Electrochemical impedance plots (Nyquist plots) of the different electrodes bare Au electrode, PEDOT/Au electrde,  rGO/PEDOT/Au electrode, Pd_nano_/rGO/PEDOT/Au electrode and BabA Ab@Pd_nano_/rGO/PEDOT/Au electrode obtained with 5 mM [Fe(CN)_6_]^3−/4−^ electrolyte containing 0.5 mM sodium phosphate buffer (pH 7.5). The frequency range is between 10 Hz to 100 kHz with amplitude of 5 mV.

To achieve both oxidation and reduction peaks, cyclic voltammetry was performed, however, square wave voltammetry and differential pulse voltammetry were performed to obtain only oxidation peaks. Figure 4A displays the CV analysis of the (a) bare Au electrode and (b) PEDOT, (c) rGO/PEDOT, (d) Pd_nano_/rGO/PEDOT and (e) BabA Ab@Pd_nano_/rGO/PEDOT-modified Au electrode. Slightly compressed redox peaks were observed for the Au electrode (curve a). Curve (b) shows the deposition of rGO, demonstrating that the material is reductive in nature. After the electropolymerization of PEDOT onto the modified electrode, there is slight improvement in the cathodic and anodic peaks (curve c). The CV curve shows the rate of electron transfer and redox peak current of the nanocomposites (Pd_nano_/rGO/PEDOT-modified Au electrode), which is significantly higher because of the good electrical conductivity achieved by combining both Pd_nano_, PEDOT and rGO^14^. Once BabA Ab was immobilized on the outer surface of the hybrid electrode, the current response decreased. The decrease in current is attributed to the fact that BabA Ab forms a layer that blocks the transfer of electrons, interrupting the redox reactions^27,28^. After analyzing the CV studies, it was revealed that the immunosensor fabrication process was effectively completed.

The charge transfer resistance (*Rct*) of the interface of the nanocomposite-coated Au electrode was monitored (Fig. 4B). The observed semicircular graph at the high-frequency region is attributed to the restricted phase of e- transfer, and the diameter is equivalent to *Rct*. The resistance of the interface to transmit charges from the electrolyte to the electrode is associated with surface variation. Therefore, a nanomaterial with a higher conductivity will have a lower resistance. Comparing the larger semicircle observed at the high-frequency region of the bare Au electrode (curve a) shows that the PEDOT and rGO/PEDOT/Au electrode exhibited a lower resistance than the bare Au electrode, because rGO and PEDOT had a higher conductivity (curves b and c). This shows the successful fabrication of rGO/PEDOT. In addition, a decrease in resistance was further observed for Pd_nano_ decorated with rGO/PEDOT on the electrode due to the high conductivity of the metal nanoparticles (curve d). The insulating Ab layer on the Pd_nano_/rGO/PEDOT/Au electrode (curve e) further blocks the transfer of electrons, resulting in a higher resistance. The BabA Ab layer on the modified electrode promotes the obstruction of electron and mass transfer. In addition, modification with BabA Ab insulates the conductive electrode surface, causing an increase in the resistance by inhibiting oxidation and reduction reactions on the electrode surface^29^. The change in the *Rct* of the electrode assembly confirms the successful immobilization of BabA Ab onto the modified Au electrode, exhibiting results that differed from the cyclic voltammetry results shown in Fig. 4A.

### Optimization of immunoassay parameters

#### Immunosensing behavior with various concentrations of the BabA antigen

**Figure 5:**
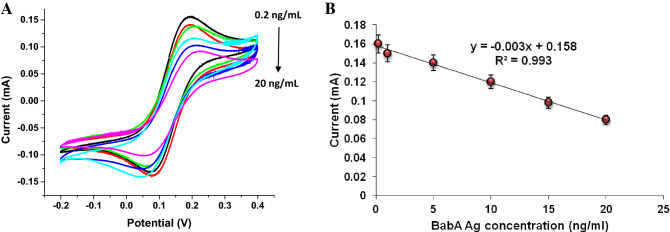
(**A**) Biosensing response studies (CV) of BabA Ab@Pd_nano_/rGO/PEDOT/Au electrode with different standard BabA Ag concentrations at 20 mVs^−1^ between -0.2 and + 0.4 V in [Fe(CN)_6_]^3−/4−^ electrolyte containing 0.5 mM sodium phosphate buffer (pH 7.5). (**B**) Standard curve of BabA Ab@Pd_nano_/rGO/PEDOT/Au electrode with different BabA Ag concentrations at 20 mVs^−1^ between -0.2 and + 0.4 V in 5 mM [Fe(CN)_6_]^3−/4−^ electrolyte containing 0.5 mM sodium phosphate buffer (pH 7.5).

Figure 5A displays the graph (CV) of various concentrations of BabA Ag added to the BabA Ab@Pd_nano_/rGO/PEDOT/Au electrode; the scanning rate was 20 mV/s, the potential ranged from − 0.2 V to + 0.4 V in PBS at a pH of 7.5 (0.5 mM), and the electrolyte was 5 mM potassium ferrocyanide/ferricyanide. As depicted in the figure, the current response decrease is proportionally to the increasing BabA Ag concentration. At the optimum concentration, Ag could interact with the Ab immobilized on the modified electrode, and the formed Ag-Ab complex serves as an inert kinetic obstacle to e- transmission in the ferricyanide mediator. As shown in the graphical image, the electrochemical response decreased with increasing the BabA Ag concentration from the lowest concentration (0.2 ng/mL) to the highest concentration (20 ng/mL), indicating that the LOD is 0.2 ng/mL (S/N = 3).

Figure 5B shows an image of a standard plot of the fabricated immunosensor based on the result obtained in Figure 5A. In the concentration range, an excellent linear correlation is exhibited. The current shift of the formed immunosensor is linear for Ag concentrations ranging from 0.2 to 20 ng/mL. The linear equation, correlation coefficient, linear range and sensitivity were calculated from the calibration curve. The sensitivity of 0.375 mA/ng mL^−1^ was calculated.

The developed BabA Ab@Pd_nano_/rGO/PEDOT/Au electrode immunosensor has shown good linearity and a low limit of detection. This sensor is more advanced than previously reported sensors^30,31^. Previously, the working range of the sensor was reported to be between 0.72–7.92 μg/mL.

#### Study of the scan rate of the immunosensor

**Figure 6:**
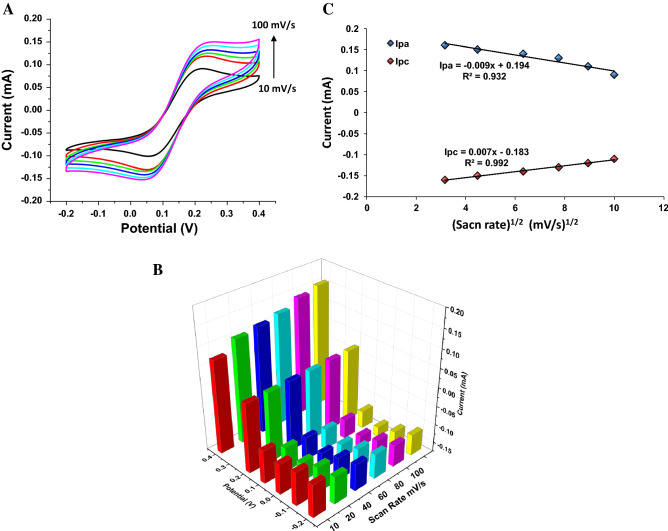
(**A**) The CV of BabA Ab@Pd_nano_/rGO/PEDOT/Au electrode scanned in [Fe(CN)_6_]^3−/4−^ electrolyte containing 0.5 mM sodium phosphate buffer (pH 7.5) with 0.2 ng/mL BabA Ag at different scan rates from 10 to 120 m V s^-1^ between -0.2 and + 0.4 V. (**B**) 3D representation of the cyclic voltammogram data of BabA Ab@Pd_nano_/rGO/PEDOT/Au electrode scanned in 5 mM [Fe(CN)_6_]^3−/4−^ electrolyte containing 0.5 mM sodium phosphate buffer (pH 7.5) with 0.2 ng/mL BabA Ag at different scan rates from 10 to 100 m V s^-1^. (**C**) The dependency of peak currents on variation of peak oxidation (I_pa_) and reduction (Ipc) as a function of square root of scan rate.

The standard CV graphs in Fig. 6A show the electrochemical studies of the modified Au electrode in PBS with a pH of 7.5 (0.5 mM), which were performed using 5 mM potassium ferrocyanide/ferricyanide as the electrolyte, 10 to 100 mV/s scanning rates and a potential range from − 0.2 V to + 0.4 V. Anodic (oxidation) and cathodic (reduction) peaks were obtained at similar potentials, while the current increased by increasing the scan rate, supporting the high stability of the immunosensor. The enhancement of the peak current with increasing the scan rate demonstrated the linearity of the diffusion-controlled mechanism. In particular, the peak-to-peak separation was enhanced with increasing the scanning frequency up to 100 mV/s.

Figure 6B represents a 3D graph of the sensor with different scanning rates. It is observed that with the increase in the scanning rate, there is also an increase in the current response. To verify the reproducibility, each experiment was replicated three times.

Figure 6C suggests that the maximum redox peak current is reached from 10 to 100 mV/s, showing the direct proportionality with the square root of the scan rate. The regression coefficient (R^2^) values and the linear formula of the peak current with respect to the square root of the scanning rate were expressed (0.932 and 0.992 for anodic and cathodic currents, respectively) for the Pd_nano_/rGO/PEDOT/Au electrode.

#### Effect of pH, temperature and time of incubation

The optimization of the diagnosing conditions was developed by conducting experiments with the immunosensor for three parameters, including pH, temperature and incubation time.

Figure S1 depicts the electrochemical performance of the immunosensor in phosphate buffers with different pH values from 6 to 8.5. The working electrode was dipped into a 5 mM potassium ferrocyanide/ferricyanide electrolyte containing 5 mL of phosphate buffer solution (with pH values ranging from 6 to 8.5) and 0.2 ng/mL BabA Ag. The current response decreased by changing the pH range from 6.0 to 7.5. The low current value represents the strong binding or optimum binding of Ag-Ab over the working electrode. Furthermore, the electrochemical response/current was increased (due to the decrease in Ag-Ab binding) with increasing the pH value up to 8.5. This occurred because of the denaturation of Ab and loss in its activity or stability due to the extremely acidic or basic surrounding. Thus, pH 7.5 was chosen as the most favorable pH for the present immunoelectrode.

Figure S2 demonstrates the effect of incubation temperature (from 10 to 40 °C) on the immunoelectrode in an electrolyte solution of 5 mM potassium ferrocyanide/ferricyanide and PBS (pH 7.5) containing 0.2 ng/mL BabA Ag. Once the temperature was increased from 10 °C to 30 °C, the current response gradually decreased, and later on, the electrochemical response increased up to 40 °C. At temperatures between 10 and 30 °C, the optimal binding of the immunocomplex (Ab-Ag binding) occurred, and thereby, the current response diminished. However, at high temperatures (more than 30 °C), the irreversible denaturation of proteins (BabA Ab and BabA Ag) may occur over the working electrode. Hence, 30 °C was selected as the optimum temperature for the present immunoelectrode.

Figure S3 shows the effect of incubation time on the immunosensor in a 5 mM potassium ferrocyanide/ferricyanide electrolyte solution containing PBS (pH 7.5) and 0.2 ng/mL BabA Ag. Once the BabA antibody on the electrode surface encounters BabA Ag in the solution with a short incubation time, the interaction of the Ag–Ab immunocomplex could not occur. As a result, the current response decreases with the increase in the incubation time up to 30 min and gradually stabilizes. After 30 min of incubation, the uniformity and similarity in the pattern of the current response (Figure S3) shows saturation in the binding of the Ag-Ab complex.

#### Real sample analysis

To perform the clinical assessment of *H. pylori,* human stool samples (n = 5) were retrieved from Bio-Diagnostics Laboratory, New Delhi. To estimate the precision, the relative standard deviation (RSD) procedure was adopted so that the BabA antigen was tested 5 times in the samples of human stools. The accuracy of the present sensor was tested using the standard approach by applying a 1.0 ng/mL standardized BabA Ag solution to the relevant samples through a recovery test. The precision was calculated within and between batch processes by a repeatability method. Within the batch processes, the analyte was added 7 times at regular intervals of 1 h, and between batch processes, the analyte was added to the incubation mixture at regular intervals of 24 h; the response was evaluated. The average recovery was estimated five times by conducting the same experiments. Consequently, a 2.1% RSD and 98.5% recovery were calculated.

**Figure 7:**
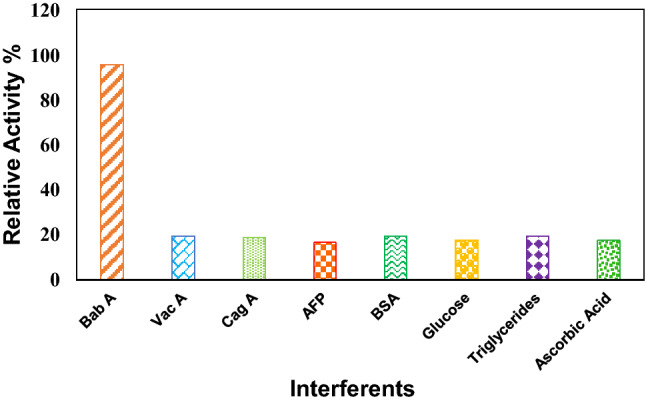
Plot of interferents studies of BabA Ab@Pd_nano_/rGO/PEDOT/Au electrode with 0.2 ng/mL BabA Ag in 0.5 mM sodium phosphate buffer (pH 7.5).

The selectivity of the immunosensor was examined with various interferents. The study was conducted using solutions of CagA, VacA, alpha-fetoprotein (AFP), BSA, glucose, triglyceride and ascorbic acid containing 1.0 ng/mL interfering compounds, and then, detection in PBS was performed by the immunosensor (pH 7.5). The findings are revealed in Fig. 7. Compared to the electrochemical response caused by BabA Ag, the current variations caused by other antigens were less than 20%. Up to the concentrations of the interfering agents, which were five times higher than those of BabA, the high specificity of the immunosensor interface was still retained.

### Storage stability and reproducibility of the BabA Ab@Pd_nano_/rGO/PEDOT/Au electrode

The shelf-life of the BabA Ab@Pd_nano_/rGO/PEDOT/Au electrode was tested with a 0.2 ng/mL standard BabA Ag solution in PBS at a regular interval of 7 days until the 9th week. A stable current value was noticed, and the activity of the immunoelectrode was calculated. The immunoelectrode preserved up to 70 percent of its activity; after it was used 20 times, the electrode was stored at 4 °C for 8–9 weeks. After testing, the electrode surface was washed with phosphate buffer solution. Figure S4 shows the decrease in the immunoelectrode activity by the denaturation of the BabA protein, while the BabA Ab@Pd_nano_/rGO/PEDOT/Au electrode's instability was retained.

The reliability of the immunosensor was evaluated by performing a reproducibility study on the BabA Ab@Pd_nano_/rGO/PEDOT/Au electrode. The reproducibility was examined by using the same concentration of BabA antigen (0.2 ng/mL) with five equally prepared working electrodes. As illustrated in Fig. S5, the 5 individually designed immunosensors had roughly identical current responses, and the interassay coefficient of variance was 5.5%. This RSD value reveals the good repeatability of the proposed immunosensor.

To summarize, we have successfully established the mechanism for constructing, optimizing and implementing a fast, quick, easy and highly selective label-free immunosensing device for sensitive *H. pylori* detection. The experiments were designed to develop a noninvasive method of *H. pylori* detection. Initially, the interaction characteristics of the electrode system were successfully determined through computational simulations. The layer-by-layer electrode assembly (Pd_nano_/rGO/PEDOT/Au electrode) generates a stable complex formation with the BabA Ag-Ab complex (− 1386.59 kJ/mol). In addition, the binding energy calculation confirms the capability of the hybrid nanomaterial-based electrode (Pd_nano_/rGO/PEDOT/Au electrode) to enhance the immunosensing performance towards the targeted biomolecule.

The modified immunosensor, as suggested by the hybrid nanomaterials, had an excellent current response to BabA Ag, which showed a broad linearity and an LOD of 0.2 ng/mL. In addition, the developed immunosensor has good specificity, stability and reproducibility. Furthermore, the application was evaluated in stool samples, showing that the sensor is easy to handle and selective for *H. pylori* diagnosis.

## Materials and methods

### Chemicals and reagents

Biologics SP-150 was used for all the electrochemical analyses (CV and impedance). 3,4-Ethylenedioxythiophene (EDOT), K_2_PdCl_4_, ethylene glycol, poly(N-vinyl-2-pyrrolidone) (PVP) and BSA were procured from Sigma Aldrich, USA. The Bab A antibody and antigens were purified at the Max von Pettenkofer Institute, Munich, Germany. Liquor ammonia, graphite, methanol, K_3_Fe(CN)_6_, and K_4_Fe(CN)_6_ were obtained from SRL, Mumbai, India. Pure 23 carat gold wire (with a 0.3 cm^2^ geometric area) was acquired from a local shop in New Delhi, India. All the solutions were prepared using deionized water (DI).

### Nanomaterial modeling

All the nanomaterial models involving the Au cluster, PEDOT, rGO and Pd_nano_ were constructed using Materials Studio^32^. The BabA antigen and antibody were designed using Discovery Studio 2017^33^ software packages (Accelrys Inc., San Diego, CA, USA). These software accurately mimic the experimental electrodes. Specifically, the Au cluster, used as an archetypical electrode, was built with its (111) facet being the most preferential orientation^34,35^ and was composed of a 56 × 49 unit cell with a C-plane orientation. On the other hand, the PEDOT polymer was constructed using the *build polymer* function provided by the software. In the simulated polymer model, the fifty PEDOT chains were built by using an optimized single chain consisting of 20 monomers (EDOT; 3,4-ethylenedioxythiophene). For rGO, the structure was initially constructed from the graphite crystal structure, which turned into a single nonperiodic layer of graphene containing 7,266 carbon atoms. According to previous experimental reports^36–38^, the ratio of carbon to oxygen of rGO is higher than that of GO, which is approximately 12:1. For this purpose, the oxygen functional groups were grafted randomly on top of the basal points of the graphene sheet layer. The rGO layer was adjusted by adding hydrogen atoms and subsequently optimized with similar protocols for the remaining layers. Furthermore, the Pd_nano_ particles were constructed with the (111) plane, which is its most energetically favorable facet according to theoretical^39,40^ and experimental studies^41^. The Pd(111) cluster was built along with the 58 × 51-unit cell and C-plane orientations to facilitate the periodic boundary box conditions with the following parameters; *a* = 160.3 Å, *b* = 140.4 Å, *c* = 259.5 Å. Subsequently, the substrate layers of the studied nanostructure were created using the *build layer* function, with a highly accurate lattice mismatch of less than 5%. For the first layer, the Au(111) substrate was used, and for the second layer, the PEDOT polymer was modified on the surface of the Au(111) cluster to build PEDOT/Au layers. Subsequently, rGO was affixed in a planar fashion onto the PEDOT/Au surface to build the rGO/PEDOT/Au layer, with rGO acting as the third layer. Finally, Pd(111) was added to the rGO/PEDOT/Au layer to computationally fabricate the complete scheme of the Pd_nano_/rGO/PEDOT/Au layer, as depicted in Fig. 1A (a).

For the biomolecules, molecular docking was essential to search the binding modes within Ag and Ab. Docking studies were performed to predict the interaction between the receptor and the ligand complex, which is somehow difficult to determine experimentally. The crystal structures of Ag and Ab were derived from the protein data bank with PDB codes of 5F7N and 5F7K, respectively^22^. For the docking simulation, Ag and Ab were selected and subsequently prepared as the input structures using ZDOCK and ZRANK modules. ZDOCK employs shape-complementary scoring methods advanced by the electrostatic and desolvation energy terms, while for ZRANK, the docked poses were reranked, resulting in an accurate bound complex structure. The best model of the largest cluster that fulfills the active binding region of Ab (Fig. 1(b)) was selected with respect to the binding energy investigations along with the nanomaterial structures.

### Metropolis Monte Carlo simulations

Metropolis Monte Carlo (MMC) adsorption studies were applied for bulk preparations. These studies were carried out to identify the lowest energy configurations of the adsorbate on the surface of the selected substrate while a gradual decrease in temperature occurred. In this analysis, the temperature was controlled automatically with three temperature cycles during the simulated annealing process. The simulated substrate and adsorbate were the electrode layers and the Ag-Ab complexes, respectively. The overall adsorption studies were performed by using the Universal force field^42,43^, which corresponds to a force field across the periodic table of elements and is reliably applied in structures having metals and organic systems^44,45^. The energy and gradient tolerances were set to 0.001 kcal/mol and 0.5 kcal/mol/Å, while the current method allocated the charges. To measure the nonbonding electrostatic and van der Waals interactions in the MMC simulations, the group-based summation process is used for the energy parameters. These parameters were applied for all the substrate layers (rather the *layer-by-layer* system) with similar adsorbates, i.e., the Ag–Ab binding pose.

The total energy of the complex was calculated as follows: after the optimum configuration of the substrate-adsorbate system was determined, the single point energy calculation was carried out using the COMPASS force field, which accurately computes the total energy of systems. Thereafter, a similar calculation was also applied for the adsorbate molecule once the substrate was removed and vice versa.

### Reduced graphene oxide (rGO) preparation

Hummer’s method was used to prepare 0.1 g of dried GO powder^46,47^; the dried GO powder was dispersed in 150 mL of DI water by sonicating for 25 to 30 min to prepare a brown uniform solution. Dropwise addition of 1.0 mL of hydrazine monohydrate to the GO solution was performed, and the resulting solution was further immersed in an oil bath (80 °C). Once immersed, the reaction mixture was stirred for 12 h. The color of the liquid changed from brown to black. Black particles settled in the solution within a few minutes. A black powder was collected in a vacuum oven at 60 °C by vacuum filtration and was further dried for 24 h^48^.

### Interface design of the immunoelectrode

#### Electropolymerization of PEDOT over the Au electrode

An alumina slurry (0.05 μM) was applied to cleaning the Au electrode, which was later immersed in a piranha solution (H_2_O_2_ (30%) and concentrated H_2_SO_4_, 3:1 proportion (v/v)) for ten minutes and subsequently cleaned by ultrasonication with DI water. The Au electrode was further cycled electrochemically in H_2_SO_4_ (1.0 M) solution until a stable gold oxide formation/reduction CV graph was achieved. The electropolymerization of EDOT on the Au electrode was carried out in an aqueous deaerated EDOT monomer solution (0.1% w/v) containing NaPSS (0.7% w/v). To completely dissolve the monomers of EDOT, the solubility of the aqueous deaerated solution should be less than the EDOT solubility (15 mM) in H_2_O at 25 °C. A cyclic voltammetry study was then performed at a scan rate of 20 mV/s for 10 cycles between − 0.9 and 1.2 V^49^.

#### Electrochemical deposition of rGO on the PEDOT-modified Au electrode

The film electrodeposition process was performed by submerging the electrode in a solution containing rGO (10 mL), and then, polymerization was achieved by applying 10 cycles from 0 V to − 1 V at a scan rate of 50 mV/s. The polymer-deposited electrode was rinsed with DI water after electropolymerization.

#### Electrochemical deposition of Pd_nano_

Pd_nano_ was deposited through an electrochemical method (cyclic voltammetry). The modified rGO/PEDOT/Au electrode was plunged into a mixture that contained 1 × 10^−3^ M K_2_PdCl_4_ supplemented with 0.5 M H_2_SO_4_. The voltage was fixed for 10 cycles (from − 0.25 V to 1.2 V at a scan rate of 50 mV/s). After the Pd_nano_/rGO/PEDOT/Au electrode was prepared, it was cleaned with DI water and air dried at room temperature.

#### BabA (antibody) immuno-interaction with the Pd_nano_/rGO/PEDOT/Au electrode

The surface of the immunoelectrode was covered by 1.0 μL of the BabA antibody (1:1000 dilution) and was then incubated at 4 °C for 12 h. The unbound antigens were removed by slowly immersing the electrode in PBS buffer (0.1 M, pH 7.5). The BabA Ab@Pd_nano_/rGO/PEDOT/Au electrode was subsequently processed with PBS containing 1% BSA for one hour to block nonreactive and nonspecific areas. Eventually, the working electrode was washed, dried and used for further characterizations and experimentation ^50^.

#### *H. pylori* detection in real samples

Five *H. pylori*-positive stool samples were collected from Bio-diagnostics Laboratory, New Delhi, India. The presence of bacterial infections in the patients was diagnosed for the 5 stool samples by a commercially available rapid urease test, and it was further confirmed by evaluation through a clinical pathologist. To prepare the stool samples, 0.1 g of stool was immersed in 0.5 ml of PBS (0.1 M, pH 7.2) and vortexed for 15 s. The suspension was centrifuged for 5 min at 5000 rpm. The formed pellets were discarded, and the clear supernatant was used in the experiment.

#### Analytical properties of the immunosensor

The LOD of the BabA Ab@ Pd_nano_/rGO/PEDOT/Au electrode is elucidated as the minimum quantity of BabA Ag required to provide the current value of the background (blank) + 3 times the SD of the blank. The LOD is calculated by using both the estimated limit of the blank (LoB) and sample test replicates known to have a low analyte concentration. The limit of quantification (LoQ) is similar to the LOD or much higher^51^. The minimum concentration required was 0.2 ng/mL.

## Supplementary information

Supplementary Information.
