# Supplementary material for: Nanohybrid-based immunosensor prepared for Helicobacter pylori BabA antigen detection through immobilized antibody assembly with @ Pdnano/rGO/PEDOT sensing platform
Source: Sci Rep. 2020 Dec 4;10:21217. doi: 10.1038/s41598-020-78068-w (PMC7719176; doi:10.1038/s41598-020-78068-w)
Supplement: Supplementary file 1 — Supplementary Information. [file 41598_2020_78068_MOESM1_ESM.doc]

**Nanohybrid-based immunosensor prepared for *Helicobacter pylori* BabA antigen detection through immobilized antibody assembly with @ Pd_nano_/rGO/PEDOT sensing platform**

*Shaivya Gupta^1^, Utkarsh Jain^1^, Bayu Tri Murti^2,3^, Athika Darumas Putri^2,3^, Ashutosh Tiwari^4,5^ and Nidhi Chauhan^1^**

*^1^Amity Institute of Nanotechnology, Amity University, Noida - 201303, Uttar Pradesh, India*

*^2^Department of Chemistry, Durban University of Technology, Durban, 4000 South Africa*

*^3^Semarang College of Pharmaceutical Sciences, Jl. Letnand Jendral Sarwo Edi Wibowo, Semarang City, 50192, Indonesia.*

*^4^Institute of Advanced Materials, IAAM, Gammalkilsvägen 18, Ulrika 590 53, Sweden*

*^5^VBRI, 7/16 Kalkaji Extn., New Delhi 110 019, India*

*email: [nchauhan1@amity.edu](mailto:ndh.chauhan1@gmail.com)

**Supplementary Figures**

| **Figure S1** | Effect of pH on the current responses of the developed BabA Ab@Pd_nano_/rGO/PEDOT/Au electrode to 0.2 ng/ml BabA Ag. |
| --- | --- |
| **Figure S2** | Effect of incubation temperature on the current responses of the developed BabA Ab@Pd_nano_/rGO/PEDOT/Au electrode to 0.2 ng/ml BabA Ag. |
| **Figure S3** | Effect of incubation time on the current responses of the developed BabA Ab@Pd_nano_/rGO/PEDOT/Au electrode to 0.2 ng/ml BabA Ag. |
| **Figure S4** | The stability of the electrochemical immunosensor BabA Ab@Pd_nano_/rGO/PEDOT/Au electrode. |
| **Figure S5** | The reproducibility of the BabA Ab@Pd_nano_/rGO/PEDOT/Au electrode was evaluated from the response to 0.2 ng/ml BabA Ag at five similar electrodes. |

**Figure S1**

**Figure S2**

**Figure S3**


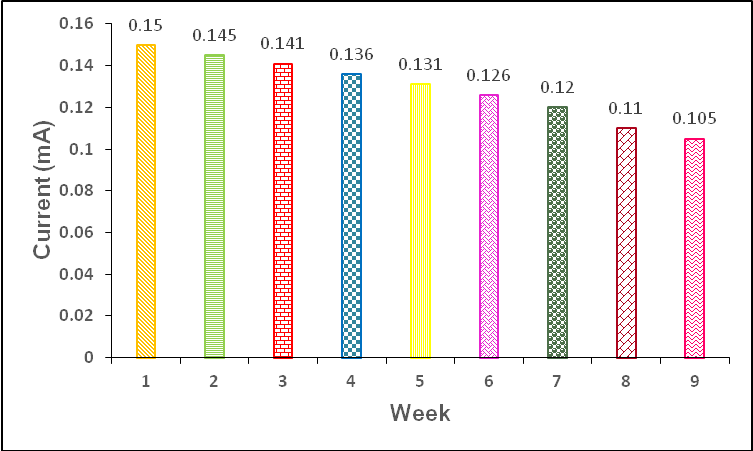


**Fig. S4**


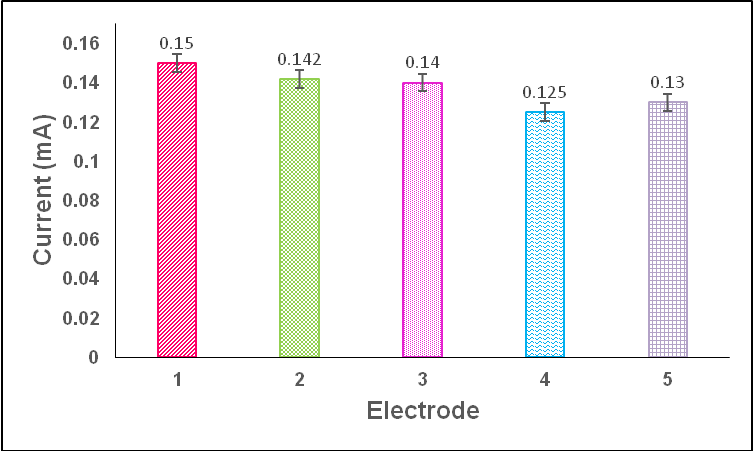


**Fig. S5**
